# Association between watching eating broadcast “Mukbang and Cookbang” and body mass index status in South Korean adolescents stratified by gender

**DOI:** 10.1186/s12937-024-00946-0

**Published:** 2024-04-18

**Authors:** Sang-yeon Park, Jeongha Eom, Sangyoun Choi, Jinhyun Kim, Eun-Cheol Park

**Affiliations:** 1 Premedical Courses, Yonsei University College of Medicine, Seoul, 03722 Republic of Korea; 2 Department of Preventive Medicine, Yonsei University College of Medicine, Seoul, Republic of Korea; 3 Institute of Health Services Research, Yonsei University, Seoul, Republic of Korea; 4 Department of Psychiatry, Yonsei University College of Medicine, Seoul, Republic of Korea; 5 Department of Preventive Medicine & Psychiatry, Institute of Health Services Research, Yonsei University College of Medicine, 50 Yonsei-ro, Seodaemun-gu, Seoul, 03722 Republic of Korea; 6 Department of Preventive Medicine & Institute of Health Services Research, Yonsei University College of Medicine, 50 Yonsei-ro, Seodaemun-gu, Seoul, 03722 Republic of Korea

**Keywords:** Mukbang, Obesity, Korea youth risk behavior survey, Adolescent, Sex difference

## Abstract

**Background:**

It has been suggested that Mukbang and Cookbang, a type of eating broadcast originating from Korea and gaining popularity, may contribute to obesity. However, despite suggestions that Mukbang might contribute to obesity, studies investigating the impact of watching Mukbang on obesity is lacking. The goal of this study is to analyze the relationship between watching Mukbang and Cookbang and body mass index (BMI) status in Korean adolescents. All analyses were stratified by gender.

**Methods:**

This study utilized data from the 2022 Korea Youth Risk Behavior Web-based Surveys. The anonymous online survey was conducted with 56,213 students, and 51,850 students (92.2%) who participated in the survey were analyzed. Participants reported the frequency of watching Mukbang and Cookbang per week over the previous 12 months. BMI was categorized into four subgroups based on percentiles: underweight (< 5th percentile), normal (5th − 85th percentiles), overweight (85th − 95th percentiles), and obese (> 95th percentile). This study used multinomial logistic regression for analysis.

**Results:**

The likelihood of being obese was significantly higher in Mukbang-watching boys compared to those who never watched Mukbang and Cookbang (adjusted odds ratio [aOR]: 1.22, 95% confidence interval [CI]: 1.12–1.32). A dose-dependent association was found between the frequency of Mukbang and Cookbang watching and the likelihood of obesity among boys (p-for-trend < 0.0001). Subgroups that currently smoke, currently drink alcohol, frequently consume fast food, or drink sweetened beverages showed significantly higher odds of being obese in the “Ever” group than those in the “Never” group.

**Conclusion:**

This study found a relationship between watching Mukbang and Cookbang and obesity in boys. Appropriate interventions should be considered for boys watching Mukbang and Cookbang.

## Background

Mukbang, a compound word of “*Meokneun*” (eating) and “*Bangsong*” (broadcasts), is a term used to refer to an “online eating show” originating from South Korea, and Cookbang refers to “online cooking show” [1]. As its name suggests, Mukbang and Cookbang (referred to as “Mukbang” from this point forward) first began in Korea [1]. Mukbang is watched online by 38% of Koreans, thus highlighting its popularity [2]. Moreover, Mukbang has spread worldwide through various international online platforms such as YouTube [3].

According to previous research, there are several potential reasons for individuals to watch Mukbang. Mukbang has gained popularity due to higher internet and social media usage by people unsatisfied in their offline lives, who engage in online activities for instant gratification and experience a feeling of “escapism” from real life [4]. In addition, people may engage in Mukbang to avoid eating alone and experiencing loneliness, drawn by its social benefits, or to derive pleasure and/or surrogate satisfaction from its content [5]. As previously mentioned, Mukbang originated in South Korea, where dining is viewed as a social activity rather than merely the act of eating. The aversion to eating alone, particularly in public places, has contributed to the popularity of watching Mukbang [6]. Through the consumption of food-related videos, individuals who eat or cook alone can experience a sense of socialization with online Mukbang streamers, helping to alleviate their feelings of loneliness [3, 7]. Moreover, the autonomous sensory meridian response provided by Mukbang can be pleasurable [5].

There are many concerns that watching Mukbang may lead to poor dietary habits and health outcomes [8]. Watching contents related to unhealthy food such as food advertisements for nutrient-poor or high-calorie foods stimulates food intake, which strongly leads obesity [9]. Especially for Mukbang, according to a previous study on the contents of Mukbang on YouTube, 83.5% of Mukbang videos contained overeating [8]. Moreover, Mukbang videos that contained overeating, food consumption within a time limit, and consumption of spicy food were each watched significantly more than those that did not contain them [8]. On the other hand, watching healthy food photography might help managing body mass index [10, 11]. Overall, through the imitation effect, Mukbang viewers consume unhealthy food, have bad eating habits, and even have eating disorder which is one of the childhood obesity comorbidities [12]. In previous study, image-based social media usage was significantly associated with BMI. Also, the study implied that ‘food porn’ has impact on rising weight among youth [13]. Furthermore, there are ongoing debates on whether watching Mukbang is addictive [14, 15]. Given the 57% increase in watching shows and films on streaming services during the coronavirus disease 2019 pandemic [16], the potential adverse effects of Mukbang could be exacerbated.

However, even though there are a few researches that investigated relationship between watching Mukbang and obesity, studies investigating the relationship in adolescents are still needed. Concurrently, obesity among South Korean adolescents has steadily increased from 15.4% in 2019 to 17.9% in 2022 [17–19]; therefore, identifying the impact of Mukbang on BMI status could be a potential policy intervention for the health status of adolescents. Hence, there is a need to analyze the relationship between BMI and watching Mukbang among Korean adolescents. Therefore, the goal of this study is to analyze the association between watching Mukbang, its frequency, and BMI in Korean adolescents by using representative cross-sectional data. Since gender differences in frequency of eating behaviors and BMI cannot be ignored [20, 21], all analyses were stratified by gender.

## Methods

### Study population and data

This study used the data acquired from the 18th Korea Youth Risk Behavior Survey (KYRBS), 2022. A question on Mukbang and Cookbang was first added in 2022. The KYRBS is a nationwide, representative, and population-based database of Korean adolescents. Based on regional characteristics, proportional stratified random sampling was carried out annually. For example, we conducted sampling based on residential circumstances and school density. An anonymous online survey was conducted with 56,213 students in approximately 400 high schools and 400 middle schools in 2022, and 51,850 students (92.2%) who participated in the survey were analyzed.

### Measures

#### BMI

We used height and weight to calculate BMI, and the percentile cutoff for BMI was used to evaluate obesity. We classified people as underweight, normal, overweight, or obese if their BMI percentile was < 5th percentile, > 5th percentile and < 85th percentile, > 85th percentile and < 95th percentile, and > 95th percentile cutoff, respectively. The percentile is based on the latest version of “Korea Growth Charts,” which was conducted in 2017 [22]. The percentile curves in the Korean National Growth Charts depict the distribution of Korean children and adolescents’ height and weight. In addition, BMI status was subdivided into severely underweight (< 3^rd^ percentile) and severely obese (> 97th percentile).

#### Mukbang and cookbang

To evaluate the frequency of Mukbang and Cookbang consumption, participants answered the question: how often they watched Mukbang and Cookbang during the previous 12 months. The answer had to be chosen from one of the following seven choices: 1-none, 2-less than once a month, 3-more than once a month and less than three times a month, 4-once or twice a week, 5-three or four times a week, 6-five or six times a week, 7-every day. We divided the participants into two groups; “Never” group consisting of individuals who answered as none (1) and “Ever” group consisting of individuals who chose other options (2–7), to observe dose-responses. In order to see if there’s any linear relationship between the odds of being obese and the frequency of watching Mukbang and Cookbang, “Ever” group was additionally divided into “Rarely” (less than once a week, options 2–3), “Occasionally” (less than 4 times a week, options 4–5), and “Always” (almost every day, options 6–7)” groups. Additionally, an analysis was conducted according to whether they were subjectively affected by watching Mukbang or Cookbang (including eating quickly, eating a lot, copying food on the show, eating snacks or late-night snacks other than mealtimes, and eating stimulatingly).

### Covariates

All covariates were appeared to significantly effect on adolescent’s BMIs according to past case-study research. Three main types of covariates were used: sociodemographic, health-related, and habitual. Age and family structure were included as socio-demographic covariates [23]. Based on which school students attended, age was divided into two groups: middle school for ages 12,13,14,15 and high school for ages 16,17,18. Based on family structure, we divided participants as having either both parents, one parent, or none. Also, the analysis was adjusted for health-related covariates including sleep duration, smoking status, alcohol use, self-reported stress levels, and physical activity [24–26]. Sleep time was divided into three groups using 6 and 8 h cutoff [27, 28]. Self-reported stress levels were classified into three groups according to the answers to “How much stress do you feel?” For physical activity, we used two variables: overall exercise and muscle strengthening exercises. Overall exercise level was divided into two groups according to the answer to the question “How many days a week do you workout over 60 min?” Overall exercise was classified as “sufficient” if it was performed for ≥ 5 days a week and “insufficient” otherwise. Muscle strengthening exercises were divided into two groups according to the answer to the question, “How many days a week do you workout to strengthen your muscles?” Muscle strengthening exercise was classified as “sufficient” if it was performed for ≥ 3 days a week and “insufficient” otherwise. Both overall and muscle-strengthening physical activities were classified according to the KYRBS guidelines. We also used academic achievement and frequency of eating fast food and sweetened beverages as habitual covariates [29]. The frequency of eating fast food was divided into two groups using the cutoff of two times a week. Frequency of eating sweetened beverage was classified as “none,” “one or two times a week,” “three or four times a week,” and “over five times a week.” Academic achievement was divided into three groups based on self-reporting questions.

### Statistical analysis

All analyses were stratified by gender. We analyze the variables using chi-square tests. To examine the association between “watching Mukbang and Cookbang” and BMI status (underweight, overweight, and obese), we used a multinomial logistic regression analysis to adjust for all covariates. The covariates are age, family structure, sweetened beverages, fast-food, alcohol status, smoking status, academic achievement, sleep duration, perceived stress level, physical activity, and muscle strengthening exercises. We also analyzed subgroups to find the relationship between the frequency of watching Mukbang and Cookbang and BMI. In addition, obese and underweight participants were divided into two groups each: severely underweight (BMI percentile < 3), underweight (3–5 percentile), obese (95–97 percentile), and severely obese (> 97 percentile) to assess any correlation between severely obese and severely underweight adolescents and watching Mukbang. Odds ratios [30] and 95% confidence intervals (CI) are presented to compare the likelihood of being underweight, overweight, and obese. Variance inflation factors for the study variables were found to be smaller than 1.32; therefore, there was no evidence of multicollinearity. We used SAS software (version 9.4; SAS Institute, Cary, North Carolina, USA) for analysis, and p-values < 0.05 were considered as statistically significant.

## Results

The study population was stratified by sex and general characteristics are presented in Table 1. In total, 50,453 participants, including 25,749 boys (51.0%) and 24,704 girls (49.0%), were used in the analysis. Among them, 63.9% of boys and 79.2% of girls watched Mukbang and Cookbang. Among participants who watch Mukbang and Cookbang, 6.9% was underweight, 11.2% was overweight, 16.7% was obese in boys, and 9.5% was underweight, 8.0% was overweight, and 9.2% was obese in girls.

**Table 1:** Health-related and socioeconomic characteristics of study participants according to the body mass index status

Variables		Boys (*N* = 25,749)									Girls (*N* = 24,704)								
		Underweight		Normal		Overweight		Obese		p-value	Underweight		Normal		Overweight		Obese		p-value
		N	(%)	N	(%)	N	(%)	N	(%)		N	(%)	N	(%)	N	(%)	N	(%)	
**Mukbang and Cookbang**										< 0.001									0.007
	Never	789	(8.5)	6219	(67.0)	933	(10.0)	1347	(14.5)		565	(11.0)	3745	(72.9)	396	(7.7)	434	(8.4)	
	Ever	1139	(6.9)	10,727	(65.2)	1846	(11.2)	2749	(16.7)		1863	(9.5)	14,329	(73.2)	1570	(8.0)	1802	(9.2)	
**Age**										< 0.001									< 0.001
	12–15	1076	(7.2)	10,061	(67.3)	1628	(10.9)	2191	(14.6)		1419	(9.8)	10,846	(74.7)	1134	(7.8)	1123	(7.7)	
	16–18	852	(7.9)	6885	(63.8)	1151	(10.7)	1905	(17.7)		1009	(9.9)	7228	(71.0)	832	(8.2)	1113	(10.9)	
**Family structure**										< 0.001									< 0.001
	Both parents	1337	(7.4)	11,799	(65.4)	1989	(11.0)	2909	(16.1)		1931	(9.7)	14,529	(73.3)	1583	(8.0)	1779	(9.0)	
	Single parent	76	(7.3)	637	(61.0)	115	(11.0)	217	(20.8)		82	(7.8)	721	(68.5)	113	(10.7)	136	(12.9)	
	No parent	515	(7.7)	4510	(67.6)	675	(10.1)	970	(14.5)		415	(10.8)	2824	(73.7)	270	(7.0)	321	(8.4)	
**Sweetened beverages**										< 0.001									< 0.001
	Non-drinker	120	(7.8)	956	(61.9)	188	(12.2)	281	(18.2)		170	(9.7)	1282	(72.9)	144	(8.2)	163	(9.3)	
	Low	487	(7.0)	4442	(64.3)	787	(11.4)	1193	(17.3)		741	(8.9)	6083	(73.3)	696	(8.4)	777	(9.4)	
	Middle	627	(7.0)	5835	(65.3)	1036	(11.6)	1434	(16.1)		764	(9.2)	6067	(73.1)	696	(8.4)	769	(9.3)	
	High	694	(8.3)	5713	(68.3)	768	(9.2)	1188	(14.2)		753	(11.9)	4642	(73.1)	430	(6.8)	527	(8.3)	
**Fast-food**										< 0.001									0.001
	Low	1334	(7.2)	11,971	(64.9)	2076	(11.3)	3067	(16.6)		1779	(9.6)	13,516	(72.9)	1525	(8.2)	1724	(9.3)	
	High	594	(8.1)	4975	(68.1)	703	(9.6)	1029	(14.1)		649	(10.5)	4558	(74.0)	441	(7.2)	512	(8.3)	
**Alcohol status**										0.001									< 0.001
	Non-drinker	1262	(8.0)	10,414	(65.9)	1670	(10.6)	2450	(15.5)		1791	(10.2)	13,029	(74.1)	1324	(7.5)	1440	(8.2)	
	Past drinker	633	(6.6)	6270	(65.7)	1069	(11.2)	1569	(16.4)		610	(8.8)	4918	(71.1)	614	(8.9)	774	(11.2)	
	Current drinker	33	(8.0)	262	(63.6)	40	(9.7)	77	(18.7)		27	(13.2)	127	(62.3)	28	(13.7)	22	(10.8)	
**Smoking status**										0.001									0.000
	Non-smoker	1753	(7.7)	14,946	(65.6)	2478	(10.9)	3593	(15.8)		2274	(9.7)	17,130	(73.4)	1846	(7.9)	2075	(8.9)	
	Current smoker	175	(5.9)	2000	(67.1)	301	(10.1)	503	(16.9)		154	(11.2)	944	(68.5)	120	(8.7)	161	(11.7)	
**Academic achievement**										< 0.001									< 0.001
	High	789	(7.7)	6975	(68.2)	1116	(10.9)	1345	(13.2)		971	(10.3)	7137	(75.9)	656	(7.0)	637	(6.8)	
	Middle	541	(7.3)	4895	(65.6)	798	(10.7)	1227	(16.4)		699	(9.1)	5735	(74.5)	613	(8.0)	652	(8.5)	
	Low	598	(7.4)	5076	(63.0)	865	(10.7)	1524	(18.9)		758	(10.0)	5202	(68.4)	697	(9.2)	947	(12.5)	
**Sleep duration**										0.015									0.179
	More than 8 h	234	(8.0)	1968	(67.4)	290	(9.9)	429	(14.7)		155	(10.2)	1100	(72.3)	126	(8.3)	140	(9.2)	
	6–8 h	750	(7.4)	6694	(66.2)	1119	(11.1)	1547	(15.3)		770	(9.6)	5866	(73.3)	681	(8.5)	687	(8.6)	
	Less than 6 h	944	(7.4)	8284	(65.1)	1370	(10.8)	2120	(16.7)		1503	(9.9)	11,108	(73.2)	1159	(7.6)	1409	(9.3)	
**Perceived stress level**										< 0.001									< 0.001
	Low	402	(7.5)	3673	(68.6)	534	(10.0)	742	(13.9)		315	(9.8)	2384	(74.2)	230	(7.2)	286	(8.9)	
	Middle	818	(7.3)	7510	(66.6)	1208	(10.7)	1734	(15.4)		967	(9.7)	7406	(74.6)	759	(7.7)	789	(8.0)	
	High	708	(7.8)	5763	(63.1)	1037	(11.4)	1620	(17.7)		1146	(9.9)	8284	(71.6)	977	(8.4)	1161	(10.0)	
**Physical activity**										< 0.001									0.025
	Insufficient	1624	(8.3)	12,581	(64.5)	2122	(10.9)	3189	(16.3)		2246	(10.0)	16,406	(73.1)	1772	(7.9)	2032	(9.0)	
	Sufficient	304	(4.9)	4365	(70.0)	657	(10.5)	907	(14.6)		182	(8.1)	1668	(74.2)	194	(8.6)	204	(9.1)	
**Muscle strengthening exercises**										< 0.001									0.003
	Insufficient	1381	(8.7)	9912	(62.3)	1770	(11.1)	2848	(17.9)		2221	(10.0)	16,174	(72.8)	1794	(8.1)	2016	(9.1)	
	Sufficient	547	(5.6)	7034	(71.5)	1009	(10.3)	1248	(12.7)		207	(8.3)	1900	(76.0)	172	(6.9)	220	(8.8)	
**Total**		1928	(7.5)	16,946	(65.8)	2779	(10.8)	4096	(15.9)		2428	(9.8)	18,074	(73.2)	1966	(8.0)	2236	(9.1)	

The results of the multinomial logistic regression analysis between watching Mukbang and Cookbang and BMI are shown in Table 2. The likelihood of obesity was found to be statistically significantly higher among the boys watching Mukbang and Cookbang (adjusted OR: 1.22, 95% CI: 1.13–1.32 in boys, adjusted OR: 1.09, CI: 0.96–1.24 in girls) after adjusting for covariates. In addition, the likelihood of overweight participants was statistically significantly higher among boys watching Mukbang and Cookbang (adjusted OR: 1.15, 95% CI: 1.04–1.26 in boys, adjusted OR: 1.06, CI: 0.93–1.21 in girls). Plus, the likelihood of underweight participants watching Mukbang and Cookbang was statistically significantly lower in both sexes (adjusted OR: 0.89, 95% CI: 0.79–0.99 in boys, adjusted OR: 0.88, CI: 0.78–0.99 in girls). Also, most covariates showed significant relationship with the odds ratio of being either obese or underweight. In boys group, odds ratio of being obese significantly increased by higher age, single parent, higher academic achievement, higher stress level, and decreased by no parent, higher frequency of drinking sweetened beverages, higher frequency of eating fast-food, sufficient muscle strengthening exercises. Odds ratio of being underweight significantly increased by higher age, and decreased by past drinker and current smoker. In girls group, odds ratio of being obese significantly increased by higher age, single parent, past drinker, higher academic achievement, and decreased by no parent and higher frequency of eating fast-food. Odds ratio of being underweight significantly increased by higher frequency of drinking sweetened beverages, current smoker, and decreased by past drinker and insufficient physical activity.

**Table 2:** Association between “watching Mukbang and Cookbang” and body mass index status

Variables		Boys (*N* = 25,749)												Girls (*N* = 24,704)											
		underweight				overweight				obese				underweight				overweight				obese			
		aOR	95% CI			aOR	95% CI			aOR	95% CI			aOR	95% CI			aOR	95% CI			aOR	95% CI		
**Mukbang and Cookbang**																									
	Never	1.000				1.000				1.000				1.000				1.000				1.000			
	Ever	0.885	(0.792	-	0.990)	1.146	1.044	-	1.259	1.221	1.130	-	1.320	0.879	0.783	-	0.987	1.061	0.932	-	1.207	1.090	0.961	-	1.236
**Age**																									
	12–15	1.000				1.000				1.000				1.000				1.000				1.000			
	16–18	1.260	1.120	-	1.417	1.067	0.963	-	1.183	1.315	1.195	-	1.448	1.041	0.938	-	1.155	1.139	1.016	-	1.276	1.442	1.287	-	1.616
**Family structure**																									
	Both parents	1.000				1.000				1.000				1.000				1.000				1.000			
	Single parent	1.005	0.758	-	1.333	1.008	0.804	-	1.265	1.267	1.050	-	1.529	0.817	0.633	-	1.054	1.305	1.034	-	1.647	1.313	1.048	-	1.644
	No parent	0.984	0.873	-	1.109	0.871	0.787	-	0.963	0.858	0.779	-	0.944	1.059	0.930	-	1.205	0.842	0.717	-	0.989	0.823	0.718	-	0.944
**Sweetened beverages**																									
	Non-drinker	1.000				1.000				1.000				1.000				1.000				1.000			
	Low	0.919	0.733	-	1.151	0.935	0.777	-	1.124	0.899	0.761	-	1.063	0.923	0.756	-	1.128	1.044	0.842	-	1.296	1.026	0.852	-	1.236
	Middle	0.877	0.690	-	1.114	0.925	0.759	-	1.126	0.809	0.689	-	0.949	0.982	0.813	-	1.187	1.008	0.818	-	1.242	0.981	0.813	-	1.183
	High	1.004	0.794	-	1.269	0.694	0.568	-	0.850	0.685	0.579	-	0.810	1.227	1.016	-	1.481	0.840	0.665	-	1.061	0.889	0.725	-	1.090
**Fast-food**																									
	Low	1.000				1.000				1.000				1.000				1.000				1.000			
	High	1.064	0.943	-	1.199	0.822	0.743	-	0.909	0.825	0.759	-	0.897	1.026	0.921	-	1.144	0.833	0.733	-	0.946	0.798	0.706	-	0.902
**Alcohol status**																									
	Non-drinker	1.000				1.000				1.000				1.000				1.000				1.000			
	Past drinker	0.884	0.783	-	0.999	1.098	0.995	-	1.212	1.030	0.940	-	1.128	0.866	0.767	-	0.977	1.197	1.058	-	1.353	1.200	1.074	-	1.341
	Current drinker	1.153	0.747	-	1.779	0.968	0.649	-	1.445	1.121	0.853	-	1.473	1.279	0.776	-	2.110	2.363	1.476	-	3.782	1.391	0.840	-	2.303
**Smoking status**																									
	Non-smoker	1.000				1.000				1.000				1.000				1.000				1.000			
	Current smoker	0.800	0.653	-	0.980	0.891	0.763	-	1.042	0.967	0.858	-	1.090	1.291	1.051	-	1.585	0.954	0.761	-	1.195	1.022	0.834	-	1.251
**Academic achievement**																									
	High	1.000				1.000				1.000				1.000				1.000				1.000			
	Middle	0.957	0.847	-	1.081	0.991	0.885	-	1.110	1.261	1.156	-	1.376	0.901	0.798	-	1.017	1.168	1.034	-	1.320	1.220	1.069	-	1.392
	Low	1.048	0.928	-	1.184	1.034	0.933	-	1.146	1.522	1.382	-	1.676	1.047	0.926	-	1.184	1.386	1.224	-	1.570	1.932	1.714	-	2.179
**Sleep duration**																									
	More than 8 h	1.000				1.000				1.000				1.000				1.000				1.000			
	6–8 h	0.885	0.741	-	1.058	1.110	0.942	-	1.307	0.953	0.832	-	1.092	0.865	0.708	-	1.056	1.036	0.822	-	1.305	0.870	0.697	-	1.086
	Less than 6 h	0.814	0.677	-	0.979	1.097	0.926	-	1.300	1.004	0.873	-	1.155	0.881	0.722	-	1.075	0.872	0.691	-	1.100	0.843	0.679	-	1.046
**Perceived stress level**																									
	Low	1.000				1.000				1.000				1.000				1.000				1.000			
	Middle	0.983	0.847	-	1.140	1.191	1.051	-	1.349	1.112	1.007	-	1.228	0.983	0.843	-	1.146	1.103	0.927	-	1.312	0.892	0.765	-	1.042
	High	1.113	0.956	-	1.296	1.344	1.176	-	1.537	1.343	1.204	-	1.499	1.017	0.877	-	1.179	1.217	1.029	-	1.441	1.138	0.982	-	1.318
**Physical activity**																									
	Insufficient	1.000				1.000				1.000				1.000				1.000				1.000			
	Sufficient	0.654	0.561	-	0.762	1.026	0.919	-	1.145	1.050	0.948	-	1.163	0.822	0.683	-	0.989	1.229	1.015	-	1.488	1.033	0.851	-	1.255
**Muscle strengthening exercises**																									
	Insufficient	1.000				1.000				1.000				1.000				1.000				1.000			
	Sufficient	0.646	0.574	-	0.727	0.808	0.737	-	0.886	0.613	0.563	-	0.667	0.878	0.728	-	1.059	0.729	0.594	-	0.894	0.944	0.783	-	1.139

*Abbreviation* aOR, adjusted odds ratio; CI, confidence interval

All variables except ‘Mukbang and Cookbang’ and ‘body mass index status’ are covariates

The results of the subgroup analyses are presented in Table 3. After dividing participants according to whether they watched Mukbang or not, the majority of subgroups showed trends similar to the main results. In particular, subgroups that currently smoke, currently drink alcohol, frequently consume fast food, or drink sweetened beverages showed significantly higher odds of being obese in the “Ever” watched Mukbang group than those in the “Never” watched Mukbang group. Among boys, students with high academic achievement or perceived stress levels were more likely to be obese when watching Mukbang.

**Fig. 1:**
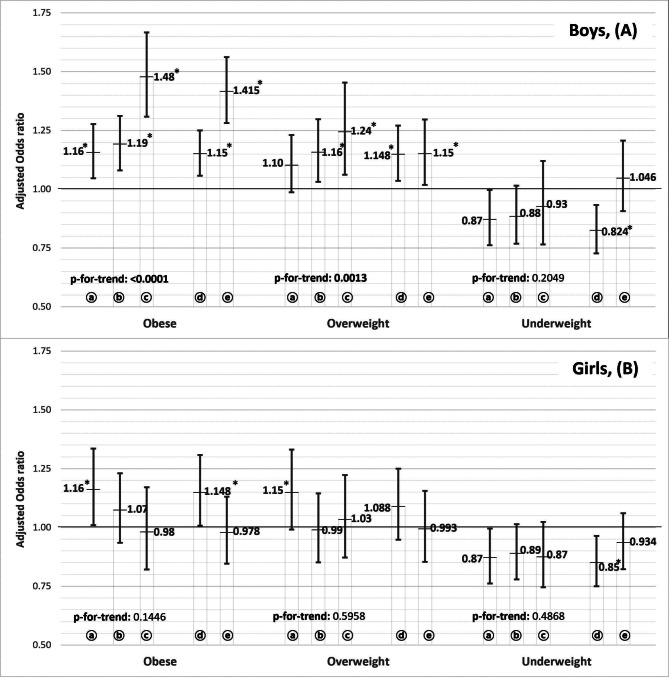
Association between “watching Mukbang and Cookbang” subdivided by frequency or subjective effect and body mass index status among boys (**A**) and girls (**B**). Rarely (less than once a week); occasionally (less than 4 times a week); always (almost every day); subjectively not affected by watching Mukbang or Cookbang; affected by watching Mukbang or Cookbang

The results of the multinomial logistic regression analysis after subgrouping those watching Mukbang and Cookbang according to their frequency and how the participants were affected is shown in Fig. 1. The current analysis indicated that even among those who watched Mukbang and Cookbang rarely, the likelihood of being underweight noticeably reduced compared to participants who never watched Mukbang in both sexes (adjusted OR: 0.87, 95% CI: 0.76–1.00 in boys, adjusted OR: 0.87, CI: 0.76–1.00 in girls). Controlling for covariates, the odds of being overweight and obese increased gradually in boys who watched Mukbang compared to those in the “Never” group as the frequency of watching Mukbang increased (p-for-trend: < 0.0001 for obese, 0.0013 for overweight). In addition, 38.6% of students watching Mukbang and Cookbang reported they are subjectively affected by the video. Among those affected, 15.4% of boys and 5.94% of girls reported they eat a lot because of watching Mukbang and Cookbang. We used chi-square test to see if it is a significant gender difference and got p-value less than 0.001. Compared to the “Never” group, the likelihood of being obese and overweight was significantly higher in boys who said they were not subjectively affected by Mukbang (adjusted OR: 1.15, 95% CI: 1.058–1.25 for obese, adjusted OR: 1.148, 95% CI: 1.036–1.271 for overweight) and in those who said they were subjectively affected (adjusted OR: 1.415, 95% CI: 1.282–1.562 for obese, adjusted OR: 1.15, 95% CI: 1.019–1.297 for overweight). There weren’t significant differences among girls.

**Table 3:** Subgroup analysis of the relationship between “watching Mukbang and Cookbang” and body mass index status

Variables		Never	Ever watching Mukbang or Cookbang											
			Underweight				Overweight				Obese			
		aOR	aOR	95% CI			aOR	95% CI			aOR	95% CI		
**Boys** (*N* = 25,749)														
**Sweetened beverages**														
	Non-drinker	1.000	1.066	0.694	-	1.635	1.143	0.796	-	1.643	1.224	0.897	-	1.671
	Low	1.000	0.920	0.744	-	1.138	1.343	1.123	-	1.608	1.136	0.975	-	1.325
	Middle	1.000	0.732	0.611	-	0.876	1.163	0.998	-	1.355	1.235	1.080	-	1.413
	High	1.000	1.003	0.826	-	1.219	0.943	0.787	-	1.131	1.319	1.140	-	1.525
**Fast-food**														
	Low	1.000	0.906	0.799	-	1.028	1.145	1.024	-	1.281	1.190	1.085	-	1.306
	High	1.000	0.833	0.686	-	1.012	1.148	0.933	-	1.412	1.319	1.115	-	1.560
**Alcohol status**														
	Non-drinker	1.000	0.860	0.752	-	0.983	1.139	1.017	-	1.275	1.211	1.097	-	1.338
	Past drinker	1.000	0.917	0.756	-	1.113	1.139	0.960	-	1.352	1.208	1.048	-	1.391
	Current drinker	1.000	2.116	0.918	-	4.875	1.634	0.691	-	3.864	2.020	1.101	-	3.705
**Smoking status**														
	Non-smoker	1.000	0.878	0.778	-	0.991	1.125	1.016	-	1.246	1.214	1.119	-	1.317
	Current smoker	1.000	0.999	0.689	-	1.448	1.334	1.005	-	1.769	1.253	0.973	-	1.613
**Academic achievement**														
	High	1.000	0.816	0.691	-	0.962	1.124	0.971	-	1.301	1.375	1.194	-	1.584
	Middle	1.000	0.990	0.804	-	1.220	1.195	0.991	-	1.440	1.144	0.987	-	1.325
	Low	1.000	0.912	0.744	-	1.118	1.139	0.963	-	1.347	1.149	1.002	-	1.317
**Perceived stress level**														
	Low	1.000	1.070	0.850	-	1.347	1.124	0.907	-	1.393	1.178	0.988	-	1.404
	Middle	1.000	0.831	0.701	-	0.985	1.126	0.975	-	1.301	1.129	1.003	-	1.270
	High	1.000	0.856	0.719	-	1.019	1.172	0.999	-	1.374	1.362	1.202	-	1.544
**Girls** (*N* = 24,704)														
**Sweetened beverages**														
	Non-drinker	1.000	0.956	0.640	-	1.429	0.670	0.447	-	1.005	0.771	0.523	-	1.138
	Low	1.000	0.850	0.698	-	1.034	1.216	0.972	-	1.521	1.199	0.977	-	1.470
	Middle	1.000	0.996	0.802	-	1.237	1.124	0.882	-	1.431	1.311	1.048	-	1.640
	High	1.000	0.776	0.634	-	0.951	0.907	0.676	-	1.216	0.851	0.661	-	1.096
**Fast-food**														
	Low	1.000	0.863	0.760	-	0.981	1.072	0.934	-	1.229	1.075	0.942	-	1.227
	High	1.000	0.945	0.741	-	1.205	1.012	0.741	-	1.380	1.118	0.834	-	1.499
**Alcohol status**														
	Non-drinker	1.000	0.940	0.826	-	1.070	1.073	0.917	-	1.256	1.010	0.866	-	1.177
	Past drinker	1.000	0.730	0.575	-	0.926	0.966	0.761	-	1.226	1.292	1.000	-	1.668
	Current drinker	1.000	0.596	0.186	-	1.906	2.909	0.785	-	10.781	1.134	0.281	-	4.574
**Smoking status**														
	Non-smoker	1.000	0.896	0.795	-	1.010	1.053	0.923	-	1.202	1.073	0.942	-	1.223
	Current smoker	1.000	0.601	0.380	-	0.950	1.130	0.629	-	2.032	1.433	0.798	-	2.575
**Academic achievement**														
	High	1.000	0.915	0.775	-	1.079	1.215	0.972	-	1.518	1.279	1.018	-	1.607
	Middle	1.000	0.810	0.653	-	1.006	1.059	0.855	-	1.311	0.983	0.787	-	1.228
	Low	1.000	0.894	0.731	-	1.093	0.889	0.712	-	1.111	1.004	0.824	-	1.223
**Perceived stress level**														
	Low	1.000	0.971	0.741	-	1.273	1.114	0.787	-	1.576	0.971	0.709	-	1.330
	Middle	1.000	0.853	0.721	-	1.008	0.997	0.813	-	1.224	1.141	0.931	-	1.400
	High	1.000	0.877	0.740	-	1.039	1.099	0.909	-	1.328	1.086	0.901	-	1.308

*Abbreviation* aOR, adjusted odds ratio; CI, confidence interval

The results regarding the association between severe obesity or underweight and watching Mukbang are shown in Table 4. In comparison with students who never watched Mukbang, boys who engaged in viewing Mukbang exhibited significantly higher odds of being severely obese and lower odds of being severely underweight (adjusted OR: 1.192, 95% CI: 1.095–1.298 for severely obese, adjusted OR: 0.856, 95% CI: 0.747–0.981 for severely underweight). No significant associations were observed among the girls.

**Table 4:** Association between “watching Mukbang and Cookbang” and severely underweight or severely obese

Variables				Severely underweight							Underweight							Obese							Severely obese							
				aOR		95% CI					aOR		95% CI					aOR		95% CI					aOR		95% CI					
	Mukbang and Cookbang																															
**Boys**		Never			1.000							1.000							1.000							1.000						
		Ever			0.856		0.747		-	0.981		0.890		0.749		-	1.058		1.220		1.039		-	1.433		1.192		1.095		-	1.298	
	**Mukbang and Cookbang**																															
**Girls**		Never			1.000							1.000							1.000							1.000						
		Ever			0.936		0.808		-	1.084		0.780		0.656		-	0.927		1.174		0.922		-	1.495		1.056		0.917		-	1.215	

*Abbreviation* aOR, adjusted odds ratio; CI, confidence interval

## Discussion

In this study, we uncovered that watching Mukbang and Cookbang was associated with significantly higher odds of obesity and lower odds of being underweight among Korean adolescent boys. Furthermore, we identified a positive dose-dependent correlation between the frequency of Mukbang consumption and obesity in boys.

Our results generally agree with those of a previous study, which revealed that the more the Korean adults watch Mukbang, the more they become obese [31, 32]. Preventing obesity in adolescents is crucial because they are prone to carrying excess weight throughout their lives [33]. In addition, obesity in adolescents is associated with psychosocial issues that stem from teasing and bullying [34]. This can contribute to depression [2]. Furthermore, boys who watched Mukbang and exhibited unhealthy eating and daily living habits such as current smoking, alcohol consumption, frequent fast-food consumption, and intake of sweetened beverages were more likely to be obese (Table 3). Given that poor habits are linked to obesity [35–37], watching Mukbang could potentially worsen obesity among adolescents. Therefore, the consideration of regulations for adolescents watching Mukbang, including limitations on viewing duration and content, is warranted. Plus, implementing nutritional education specific to Mukbang could serve as an alternative intervention. Nutritional education for children and adolescents is effective in reducing BMI [38] and facilitating a permanent change of one’s eating habit [39]. Educating the fact that overeating and consuming a lot of provocative food may cause undesirable health issues may provide significant help in blocking the casual link between watching Mukbang and Cookbang and bad eating habits.

The mechanism linking Mukbang watching to obesity remains unclear; however, we developed several hypotheses. First, we propose the possibility of obesity resulting from behaviors, such as eating quickly, consuming large quantities, mimicking foods seen on the show, snacking, having late-night snacks outside of regular mealtimes, and engaging in stimulating eating habits. Of the students who watched Mukbang, 38.6% reported being subjectively affected. In our study, boys who were subjectively affected had a significantly higher likelyhood of being obese, even when compared with boys who were not subjectively affected by Mukbang. We also suggest that watching Mukbang might contribute to obesity by influencing the biochemical mechanisms in the body and promoting appetite. Individuals who watch Mukbang and Cookbang may experience an increase in blood ghrelin secretion, leading to an elevated appetite and increased food consumption [40]. Additionally, watching Mukbang could contribute to obesity by reducing engagement in other activities, including exercise and social interaction. Individuals watching Mukbang engage in more screen time and show higher tendency of being obese [41, 42].

There were noticeable gender differences in the results regarding obesity based on Mukbang watching, with the differences being more pronounced in boys than in girls. After consuming food-related content, girls experienced more stress regarding eating than boys [43]. Similarly, girls tended to have more food-related conflicts than boys and may perceive lesser need to eat [44]. Also, adolescent girls are more influenced by media, parents, and peers to be thin [45]. Meanwhile, people who try to lose their weight have lower appetite after watching Mukbang than people who don’t [46]. Therefore, when girls feel the urge to eat after watching Mukbang and Cookbang, they tend to suppress the urge more strongly than boys. This can be validated by KYRBS survey data. Among students who responded their eating habits are affected by watching Mukbang and Cookbang, 15.4% of boys and 5.94% of girls responded watching the videos made themselves eat a lot. Thus, we can conclude that boys are more vulnerable to the effect of watching Mukbang and Cookbang on obesity than girls.

It is also plausible that obese students were more inclined to watching Mukbang, as they tended to be more attracted to food and eating stimuli than their lean counterparts [47]. For instance, they may be more prone to consuming late-night snacks or engaging in stimulating eating habits and actively seek Mukbang content to share eating experiences or ideas. Conversely, a prior study that conducted a “food porn” experiment revealed that participants who viewed food photography a few times expressed an increased desire for food, but with more repetitions, their desire for food diminished compared to the initial levels [48]. Therefore, additional research is needed to ascertain the causal relationship between watching Mukbang and obesity, along with a thorough examination of its underlying mechanisms.

There are several limitations in this study. Given that the KYRBS relies on cross-sectional data, establishing a cause-and-effect relationship and directly attributing the impact of watching Mukbang to BMI is challenging. Moreover, reliance on self-reported data likely introduces a decline in data accuracy. As a result, some results in Table 2 were inconsistent with previous studies. For example, boys and girls in this study who consumed sweetened beverages or fast food a lot showed higher odds of being obese than those who didn’t. The lack of an objective scale for measuring eating habits and physical activity may result in lower reliability. Intake of foods and meals especially on snacks are frequently under-reported [49]. Furthermore, obese people whose BMIs are not less than 30 under-report their intake [50]. Because of these problems, obese boys and girls might report they ate less sweetened beverages or fast food than they actually did. Therefore, there were results in Table 2 that boys and girls in this study who reported they rarely consumed sweetened beverages or fast food showed higher odds of being obese than those who didn’t, which are inconsistent with previous studies. Additionally, the study was unable to assess specific details about Mukbang, such as the content watched, timing of Mukbang viewership, or the activities adolescents engaged in while watching Mukbang. Finally, our findings may not apply to other countries with distinct cultures.

## Conclusion

This study revealed that boys who watch Mukbang suffer from obesity, showing a dose-dependent relationship, particularly those with poor eating and daily living habits. Since the positive linear relationship between the odds of being obese and BMI was revealed in boys group, restricting the frequency of watching Mukbang will significantly help reduce their chances of becoming obese. Appropriate interventions should thus be considered for boys watching Mukbang. The government and education institutions may need some interventions, such as regulating watching or broadcasting eating show, informing the potential risk of watching Mukbang, and implementing nutritional education, to promote the health status of adolescent boys.

## Data Availability

The data analyzed in this study were sourced from the publicly accessible 2022 Korea Youth Risk Behavior Web-based Survey (KYRBS. All data were retrieved from the official KYRBS website (https://www.kdca.go.kr/yhs/).

## Abbreviations

BMI: body mass index
aOR: adjusted odds ratio
CI: confidence interval, KYRBS Korea Youth Risk Behavior Web-based Survey

## References

[1] Cho E-H (2020). A study on the Trend and the Cultural Phenomenon of Mukbang. J Korea Contents Association.

[2] KOBACO, 2019 Media & Consumer Research. 2019, KOBACO: Seoul. p. 106.

[3] Strand M, Gustafsson SA, Culture (2020). Mukbang and disordered eating: a netnographic analysis of online eating broadcasts. Med Psychiatry.

[4] Sanskriti S (2023). The spectrum of motivations behind watching mukbang videos and its Health effects on its viewers: a review. Cureus.

[5] Anjani L et al. *Why do people watch others eat food? An Empirical Study on the Motivations and Practices of Mukbang Viewers*. in *Proceedings of the* 2020 *CHI conference on human factors in computing systems*. 2020.

[6] Jackson K. *Inside ‘mukbang’: How some professional binge-eaters earn thousands. Today*. 2018.

[7] Choe H (2019). Eating together multimodally: collaborative eating in mukbang, a Korean livestream of eating. Lang Soc.

[8] Kang E (2020). The popularity of eating broadcast: content analysis of Mukbang YouTube videos, media coverage, and the health impact of mukbang on public. Health Inf J.

[9] Boulos R (2012). ObesiTV: how television is influencing the obesity epidemic. Physiol Behav.

[10] Andersen T, Byrne DV, Wang QJ (2021). How digital food affects our analog lives: the impact of food photography on healthy eating behavior. Front Psychol.

[11] Seal A, Gavaravarapu SM, Konapur A (2022). Can foodporn prime healthy eating? Thinking beyond digital gazing and satiety. Eur J Clin Nutr.

[12] Lister NB (2023). Child and adolescent obesity. Nat Rev Dis Primers.

[13] Chauhan J, Hedaoo R, Patil M. Gastroporn on Social Media and Its Association with Food choices and Body Mass Index among Youth. Volume 14. Advanced Studies in Biology; 2022. pp. 137–51. 1.

[14] Kircaburun K (2021). Development and validation of the mukbang addiction scale. Int J Mental Health Addict.

[15] Kircaburun K (2021). Problematic mukbang watching and its relationship to disordered eating and internet addiction: a pilot study among emerging adult mukbang watchers. Int J Mental Health Addict.

[16] Kemp S. *Digital 2020 April Global Statshot Report*. 2020 [cited Dec 2nd, 2023; https://datareportal.com/reports/digital-2020-april-global-statshot.

[17] Jha S, Mehendale AM. Increased incidence of obesity in children and adolescents post-COVID-19 pandemic: a review article. Cureus, 2022. 14(9). 10.7759/cureus.29348 PMC9582903 36284800

[18] Jenssen BP et al. COVID-19 and changes in child obesity. Pediatrics, 2021. 147(5). 10.1542/peds.2021-050123 33653879

[19] Byeon H (2022). Predicting South Korean adolescents vulnerable to obesity after the COVID-19 pandemic using categorical boosting and shapley additive explanation values: a population-based cross-sectional survey. Front Pead.

[20] VanKim NA (2019). Gender expression and sexual orientation differences in Diet Quality and Eating habits from Adolescence to Young Adulthood. J Acad Nutr Diet.

[21] Kim M-H (2012). Eating habits, self perception of body image, and weight control behavior by gender in Korean adolescents-using data from a 2010 Korea Youth Risk Behavior web-based Survey. J East Asian Soc Diet Life.

[22] Kim JH (2018). The 2017 Korean National Growth Charts for children and adolescents: development, improvement, and prospects. Korean J Pediatr.

[23] Kautiainen S (2009). Sociodemographic factors and a secular trend of adolescent overweight in Finland. Int J Pediatr Obes.

[24] Ajibewa TA (2020). Adolescent stress: a predictor of dieting behaviors in youth with overweight/obesity. Appetite.

[25] Zeller MH (2016). Associations among excess weight status and tobacco, alcohol, and illicit drug use in a large national sample of early adolescent youth. Prev Sci.

[26] Reichert FF (2009). Physical activity as a predictor of adolescent body fatness: a systematic review. Sports Med.

[27] Duraccio KM (2019). Poor sleep and adolescent obesity risk: a narrative review of potential mechanisms. Adolesc Health Med Ther.

[28] Sayin FK, Buyukinan M (2016). Sleep duration and media Time have a major impact on insulin resistance and metabolic risk factors in obese children and adolescents. Child Obes.

[29] Benazeera UJ (2014). Association between eating habits and body mass index (BMI) of adolescents. Int J Med Sci Public Health.

[30] Stea TH, Torstveit MK (2014). Association of lifestyle habits and academic achievement in Norwegian adolescents: a cross-sectional study. BMC Public Health.

[31] Yeon K (2023). Health threats of new social media trends: the effects of frequent mukbang watching on overweight and obesity. Appl Econ Lett.

[32] Yoo SW, Shin GH, Kim SJ (2021). Does Mukbang watching really affect obesity? Focusing on the factors related to Health and Mukbang watching. Korean J Journalism Communication Stud (KJJCS).

[33] Simmonds M (2016). Predicting adult obesity from childhood obesity: a systematic review and meta-analysis. Obes Rev.

[34] Harriger JA, Thompson JK (2012). Psychological consequences of obesity: weight bias and body image in overweight and obese youth. Int Rev Psychiatry.

[35] Seema S (2021). Prevalence and contributing factors for adolescent obesity in present era: cross-sectional study. J Family Med Prim Care.

[36] Jacobs M (2019). Adolescent smoking: the relationship between cigarette consumption and BMI. Addict Behav Rep.

[37] Liberali R (2021). Latent class analysis of lifestyle risk factors and association with overweight and/or obesity in children and adolescents: systematic review. Child Obes.

[38] da Silveira JAC (2013). The effect of participation in school-based nutrition education interventions on body mass index: a meta-analysis of randomized controlled community trials. Prev Med.

[39] Pietrzak A (2020). Childhood obesity in a Psychosocial Perspective. The role of Nutrition Education. Edukacja Elementarna w Teorii i Praktyce.

[40] Schüssler P (2012). Ghrelin levels increase after pictures showing food. Obes (Silver Spring).

[41] Tambalis KD (2018). Insufficient sleep duration is Associated with Dietary habits, screen time, and obesity in children. J Clin Sleep Med.

[42] Fang K (2019). Screen time and childhood overweight/obesity: a systematic review and meta-analysis. Child Care Health Dev.

[43] Tazeoğlu A, BOZDOGAN FBK (2022). The effect of watching food videos on social media on increased appetite and food consumption. Nutrición Clínica Y Dietética Hospitalaria.

[44] Rolls BJ, Fedoroff IC, Guthrie JF (1991). Gender differences in eating behavior and body weight regulation. Health Psychol.

[45] McCabe MP, Ricciardelli LA (2001). Parent, peer, and media influences on body image and strategies to both increase and decrease body size among adolescent boys and girls. Adolescence.

[46] Xu W. *Does watching mukbangs help you diet? The effect of the mukbang on the desire to eat* 2019.

[47] Cinciripini PM (1984). Food choice and eating behavior among obese, lean, and normal individuals. Behav Modif.

[48] Andersen T, Byrne DV, Wang QJ. How Digital Food affects our Analog lives: the Impact of Food Photography on Healthy Eating Behavior. Frontiers in Psychology; 2021. p. 12. 10.3389/fpsyg.2021.634261 PMC8056120 33889111

[49] Gemming L, Ni C, Mhurchu (2016). Dietary under-reporting: what foods and which meals are typically under-reported?. Eur J Clin Nutr.

[50] Wehling H, Lusher J (2019). People with a body mass index ⩾30 under-report their dietary intake: a systematic review. J Health Psychol.

